# International Validation of Echocardiographic Artificial Intelligence Amyloid Detection Algorithm

**DOI:** 10.1016/j.jacadv.2025.102067

**Published:** 2025-09-17

**Authors:** Grant Duffy, Evangelos K. Oikonomou, Nicholas Easton, Hiroki Usuku, Jigesh Patel, Yoshinori Katsumata, Daisuke Yamasawa, Lily Stern, Shinichi Goto, Kenichi Tsujita, Paul Cheng, Rohan Khera, Faraz S. Ahmad, David Ouyang

**Affiliations:** a Smidt Heart Institute, Cedars-Sinai Medical Center, Los Angeles, California, USA; b Section of Cardiovascular Medicine, Department of Internal Medicine, Yale School of Medicine, New Haven, Connecticut, USA; c Cardiovascular Data Science (CarDS) Lab, Yale School of Medicine, New Haven, Connecticut, USA; d Department of Medicine, Northwestern Medicine, Chicago, Illinois, USA; e Department of Cardiovascular Medicine, Graduate School of Medical Sciences, Kumamoto University, Chuo-ku, Kumamoto, Japan; f Department of Cardiology, Keio University School of Medicine, Shinjuku, Tokyo, Japan; g Department of Medicine, Tokai University School of Medicine, Isehara, Kanagawa, Japan; h One Brave Idea, Division of Cardiovascular Medicine, Brigham and Women’s Hospital, Boston, Massachusetts, USA; i Division of Cardiology, Department of Medicine, Stanford University, Palo Alto, California, USA

**Keywords:** artificial intelligence, cardiac amyloidosis, computer vision, echocardiography

## Abstract

**Background:**

Diagnosis of cardiac amyloidosis (CA) is often missed or delayed due to confusion with other causes of increased left ventricular wall thickness. Conventional transthoracic echocardiographic measurements like global longitudinal strain have shown promise in distinguishing CA, but with limited specificity.

**Objectives:**

We conducted a multisite retrospective case-control study to investigate the performance of a computer vision algorithm for CA identification across multiple international sites.

**Methods:**

EchoNet-left ventricular hypertrophy (LVH) is a computer vision deep learning algorithm for the detection of CA based on parasternal long axis and apical-4-chamber view videos. We evaluated EchoNet-LVH’s ability to distinguish between the echocardiogram studies of 574 CA patients and 979 controls. We reported discrimination performance with an area under the receiver operating characteristic curve and associated sensitivity, specificity, and positive predictive value at the prespecified threshold.

**Results:**

EchoNet-LVH had an area under the receiver operating characteristic curve of 0.896 (95% CI: 0.875-0.916). At the prespecified model threshold optimizing for specificity, EchoNet-LVH had a sensitivity of 0.644 (95% CI: 0.601-0.685), specificity of 0.988 (95% CI: 0.978-0.994), positive predictive value of 0.968 (95% CI: 0.944-0.984), and negative predictive value of 0.828 (95% CI: 0.804-0.850). There was no evidence of heterogeneity in performance by site, race, sex, age, body mass index, CA subtype, or ultrasound manufacturer.

**Conclusions:**

EchoNet-LVH can assist with earlier and accurate diagnosis of CA. EchoNet-LVH achieved development goals to be highly specific to maximize positive predictive value of downstream confirmatory testing since CA is a rare disease.

Cardiac amyloidosis (CA) is caused by deposition of misfolded proteins in the myocardium, including transthyretin or immunoglobulin light chains.^1^^,^^2^ Regardless of the etiology, CA leads to increased left ventricular wall thickness and heart failure; however, early symptoms can be nonspecific and not readily recognized.^3^, ^4^, ^5^ Common echocardiographic measurements are insufficient to precisely discriminate CA from other etiologies of left ventricular hypertrophy (LVH) or heart failure.^6^, ^7^, ^8^

There is concern that CA is underdiagnosed and diagnosed too late, which limit the opportunity to receive recent targeted therapies that improve quality of life and decrease mortality outcomes in CA patients.^9^, ^10^, ^11^ Recent research has focused on methods that can assist with early identification of CA.^12^, ^13^, ^14^ Echocardiography is one of the most common initial tests when evaluating patients with heart failure symptoms, with typical CA features including increased left ventricular wall thickness, normal or small left ventricular cavity, biatrial enlargement, preserved left ventricular ejection fraction, and diastolic dysfunction.^1^^,^^2^ However, many of these features are also commonly found in other forms of heart failure with preserved ejection fraction and have limited specificity to identify CA,^6^^,^^8^ resulting hesitancy to highlight suspicion for CA.

Recent advances in computer vision and artificial intelligence (AI) have enabled precision phenotyping of structure and function in cardiac ultrasound as well as other forms of cardiovascular diagnostics.^15^, ^16^, ^17^, ^18^, ^19^ AI applied to echocardiography can precisely estimate wall thickness,^13^ assess mitral regurgitation severity,^20^ and left ventricular ejection fraction^21^ as well as detect CA,^13^ hypertrophic cardiomyopathy, and diastolic dysfunction.^22^ EchoNet-LVH is one such computer vision model developed for the detection of CA.^13^ An automated pipeline, EchoNet-LVH identifies parasternal long-axis and apical-4-chamber views from transthoracic echocardiogram studies to precisely measure the wall thickness and identify texture and motion suggestive of CA. Trained against other forms of LVH as controls against CA cases, EchoNet-LVH was designed to be specific in discriminating between CA and phenotypic mimics. This model was “frozen” and not further fine-tuned to perform further analyses and external validation.

In this study, we evaluated the performance EchoNet-LVH across multiple new health care systems on videos that the model has never seen before (Central Illustration). We conducted a multisite international retrospective case-control study evaluating EchoNet-LVH’s ability to distinguish between the echocardiogram studies of CA patients and controls. We reported discrimination performance with area under the receiver operating characteristic curve (AUC) and associated sensitivity, specificity, and positive predictive value (PPV) at a prespecified threshold to identify CA.

**Central Illustration:**
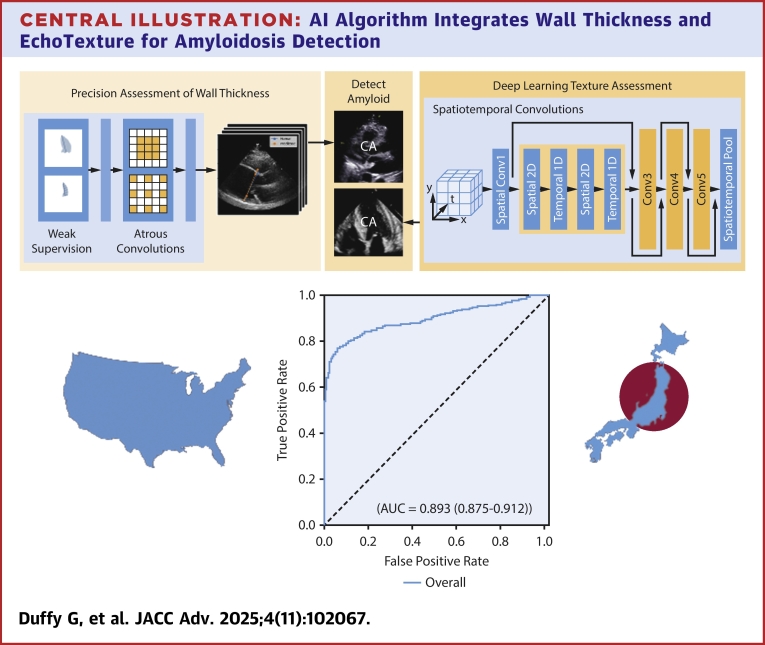
AI Algorithm Integrates Wall Thickness and EchoTexture for Amyloidosis Detection A fully automated computer vision algorithm identifies key features from the parasternal long axis and apical-4-chamber echocardiogram videos to identify cardiac amyloidosis from visual features. This algorithm was evaluated across five geographically and ethnically diverse cohorts across the United States of America and Japan to show strong generalizable performance across a range of ultrasound vendors and patient demographics. AUC = area under the receiver operating characteristic curve; CA = cardiac amyloidosis.

## Methods

### Study design

We conducted an international multicenter retrospective case-control cohort study with participants from multiple geographically distinct health care systems to evaluate EchoNet-LVH. EchoNet-LVH was previously developed using CA cases and controls from Stanford Health care,^20^ so this study serves as geographically distinct external validation. Patients were sourced from Cedars-Sinai Medical Center in Los Angeles; California, Keio University in Tokyo, Japan; Northwestern Medicine in Chicago, Illinois; Kumamoto University in Kumamoto; and Yale-New Haven Hospital in New Haven, Connecticut. A total of 574 patients were identified as having CA and matched to 979 randomly selected controls from patients receiving echocardiography and at least 65 years of age. CA patients were diagnosed with transthyretin (ATTR), light chain (AL) amyloidosis, or other forms of CA using a combination of nuclear CA imaging (including single photon emission computed tomography with approved bone radiotracers, such as technetium-99m pyrophosphate), monoclonal gammopathy testing, genetic testing, and/or tissue biopsy. The controls either had negative testing for CA or testing was not performed. Given the low population prevalence of CA, the likelihood of undiagnosed CA in the controls was thought to be minor and unlikely to change the analysis. This study was approved by the Cedars-Sinai Institutional Review Board.

### Computer vision model

EchoNet-LVH’s development approach and internal validation has been previously described.^20^ In short, EchoNet-LVH is an automated machine learning pipeline that automatically identifies parasternal long axis and apical-4-chamber views from transthoracic echocardiogram studies, precisely measures wall thickness, and assesses texture and motion from the apical-4-chamber view echocardiogram videos to assess suspicion for CA. Information from the apical-4-chamber view is synthesized with a segmentation model’s assessment of wall thickness from the parasternal long-axis videos to come up with a suspicion for CA. Given the low population prevalence of CA, a prespecified threshold (0.8) in the summative assessment was chosen to optimize and prioritize for specificity, which in turn, maximizes PPV. Echocardiogram videos were obtained in DICOM format and a fully automatic pipeline analyzed the study. All sites ran the model independently without transfer of imaging data through the sharing of a Docker container with a code for the full end-to-end pipeline.

### Statistical analysis

Continuous variables were reported using median with 25th to 75th percentiles (Q1-Q3), and categorical variables were reported with number (percentage). Performance of the model for discriminating CA was evaluated using AUC, sensitivity, specificity, PPV, and negative predictive value with 95% CIs. AUC was calculated comparing ground truth labels and model output, both for overall AUC and for subgroup analysis. Statistical analysis was performed in Python (Python Software Foundation).

## Results

Demographics and clinical characteristics of the study cohort are shown in Table 1. The median age of the cohort was 78 years (Q1-Q3: 72-84) and 77.3% male, with cases matched 2:1 with controls. In the cases, there was representation from both AL (29.4%) and ATTR (67.8%). There was a wide range of age, body mass index, and ultrasound manufacturers across the sites with subgroup analysis to evaluate for heterogeneity of EchoNet-LVH performance.

**Table 1:** Patient Characteristics

Age, y	78 (72-84)
Men	1,200 (77.3%)
Cardiac amyloidosis	574 (36.9%)
Intraventricular septum, cm	1.27 (1.02-1.48)
Subtype	
AL	169 (29.4%)
ATTR	389 (67.8%)
Other	16 (2.8%)
Age, y	
<74	515 (33.2%)
75-84	673 (43.3%)
>85	365 (23.5%)
BMI, kg/m^2^	
<25	695 (44.8%)
25-30	538 (34.6%)
>30	313 (20.2%)
Ultrasound manufacturer	
Philips	1,065 (68.6%)
GE	187 (12.0%)
Siemens	130 (8.4%)
Vingmed	96 (6.2%)
Toshiba	66 (4.2%)
Fujifilm	9 (0.6%)

Values are median (Q1-Q3) or n (%).

AL = light chain amyloidosis; ATTR = transthyretin amyloidosis; BMI = body mass index; GE = general electric.

**Figure 1:**
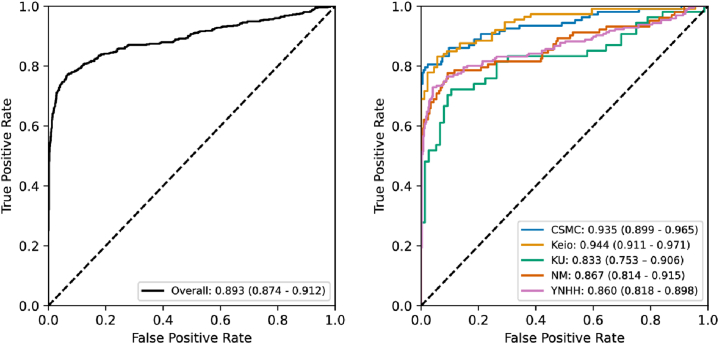
Receiver Operating Characteristic Curve for EchoNet-LVH Overall performance of AI algorithm and subpopulation performance by site. CSMC = Cedars-Sinai Medical Center; KU = Kumamoto University; NU = Northwestern University; YNHH = Yale New Haven Hospital.

EchoNet-LVH had an overall AUC of 0.893 (95% CI: 0.874-0.912) with minimal site level variation in performance (Figure 1, Table 2). The lowest site AUC was Kumamoto University with an AUC 0.833 (95% CI: 0.753-0.906) and the highest site AUC was Keio University with an AUC of 0.944 (95% CI: 0.911-0.971). The overall sensitivity was 0.648 (95% CI: 0.607-0.687) and the overall specificity was 0.982 (95% CI: 0.971-0.989). There was no significant heterogeneity in other performance characteristics across site. At a 2:1 ratio of controls to cases, EchoNet-LVH had a PPV of 0.954 (95% CI: 0.928-0.972) and a negative predictive value of 0.826 (95% CI: 0.804-0.848).

**Table 2:** Overall Performance

	N	Sensitivity (95% CI)	Specificity (95% CI)	PPV (95% CI)	NPV (95% CI)	AUROC (95% CI)
Overall	1,553	0.648 (0.607-0.687)	0.982 (0.971-0.989)	0.954 (0.928-0.972)	0.826 (0.803-0.848)	0.893 (0.874-0.912)
CSMC	324	0.648 (0.550-0.738)	1.000 (0.983-1.000)	1.000 (0.949-1.000)	0.850 (0.800-0.892)	0.935 (0.899-0.965)
Keio	202	0.779 (0.691-0.851)	0.966 (0.905-0.993)	0.967 (0.907-0.993)	0.775 (0.686-0.849)	0.944 (0.911-0.971)
NM	309	0.621 (0.520-0.715)	0.981 (0.951-0.995)	0.941 (0.856-0.984)	0.838 (0.785-0.882)	0.867 (0.814-0.915)
YNHH	588	0.577 (0.504-0.647)	0.990 (0.974-0.997)	0.966 (0.915-0.991)	0.824 (0.786-0.857)	0.860 (0.818-0.898)
KU	130	0.685 (0.544-0.805)	0.908 (0.819-0.962)	0.841 (0.699-0.934)	0.802 (0.702-0.880)	0.833 (0.753-0.906)

AUROC = area under the receiver operating characteristic curve; CSMC = Cedars-Sinai Medical Center; KU = Kumamoto University; NPV = negative predictive value; NU = Northwestern University; PPV = positive predictive value; YNHH = Yale New Haven Hospital.

EchoNet-LVH also had similar performance across patient characteristics and ultrasound manufacturer (Table 3). EchoNet-LVH had an AUC of 0.907 (95% CI: 0.862-0.940) for detecting AL CA and an AUC of 0.890 (95% CI: 0.867-0.914) for detecting ATTR CA. Our model had similar performance for men (AUC of 0.894 [95% CI: 0.876-0.910]) and women (AUC of 0.872 [95% CI: 0.808-0.940]). There was no significant heterogeneity by race, age, body mass index, or ultrasound manufacturer. Across all key groups, there was similar sensitivity and specificity of our approach, and there was no trend for differences in performance across subclasses.

**Table 3:** Subgroup Performance

	n	Sensitivity (95% CI)	Specificity (95% CI)	PPV (95% CI)	NPV (95% CI)	AUROC (95% CI)
Sex						
Male	1,200	0.665 (0.621-0.707)	0.979 (0.966-0.988)	0.955 (0.928-0.975)	0.812 (0.785-0.838)	0.894 (0.876-0.910)
Female	353	0.556 (0.447-0.660)	0.989 (0.967-0.998)	0.943 (0.843-0.988)	0.867 (0.823-0.903)	0.872 (0.808-0.940)
Race						
White	888	0.578 (0.515-0.639)	0.990 (0.979-0.996)	0.961 (0.918-0.986)	0.851 (0.823-0.876)	0.871 (0.837-0.902)
Asian	332	0.749 (0.676-0.812)	0.939 (0.891-0.971)	0.926 (0.868-0.964)	0.787 (0.723-0.842)	0.904 (0.866-0.934)
Black	174	0.642 (0.543-0.732)	0.985 (0.921-1.000)	0.986 (0.922-1.000)	0.638 (0.539-0.730)	0.884 (0.831-0.931)
Hispanic	66	0.688 (0.413-0.890)	1.000 (0.929-1.000)	1.000 (0.715-1.000)	0.909 (0.800-0.970)	0.963 (0.894-1.000)
Other	93	0.704 (0.498-0.862)	0.985 (0.918-1.000)	0.950 (0.751-0.999)	0.890 (0.795-0.951)	0.880 (0.762-0.973)
Age, y						
<75	515	0.677 (0.607-0.741)	0.978 (0.955-0.991)	0.950 (0.900-0.980)	0.829 (0.787-0.866)	0.918 (0.892-0.953)
75-84	673	0.629 (0.563-0.692)	0.985 (0.967-0.994)	0.960 (0.915-0.985)	0.821 (0.784-0.855)	0.874 (0.837-0.908)
85+	365	0.643 (0.549-0.731)	0.991 (0.968-0.999)	0.974 (0.908-0.997)	0.845 (0.796-0.887)	0.903 (0.860-0.941)
BMI, kg/m^2^^a^						
<25	695	0.672 (0.595-0.714)	0.980 (0.959-0.992)	0.939 (0.898-0.967)	0.796 (0.757-0.831)	0.881 (0.853-0.904)
25-30	538	0.650 (0.583-0.721)	0.987 (0.968-0.997)	0.970 (0.925-0.992)	0.827 (0.786-0.862)	0.905 (0.877-0.932)
>30	313	0.556 (0.447-0.689)	1.000 (0.985-1.000)	0.976 (0.871-0.999)	0.882 (0.838-0.918)	0.883 (0.824-0.941)
Amyloid subtype						
AL	1,072	0.669 (0.592-0.739)	0.967 (0.945-0.982)	0.911 (0.847-0.955)	0.941 (0.924-0.969)	0.907 (0.862-0.940)
ATTR	1,292	0.640 (0.590-0.688)	0.988 (0.970-0.997)	0.958 (0.926-0.979)	0.864 (0.842-0.885)	0.890 (0.867-0.914)
Other	995	0.625 (0.354-0.848)	0.996 (0.977-1.000)	0.357 (0.186-0.559)	0.994 (0.987-0.998)	0.932 (0.857-0.995)
Ultrasound Manufacturer						
Philips	1,065	0.605 (0.554-0.654)	0.982 (0.969-0.991)	0.951 (0.916-0.975)	0.813 (0.785-0.839)	0.885 (0.859-0.909)
GE Ultrasound	187	0.741 (0.610-0.847)	0.977 (0.934-0.995)	0.935 (0.821-0.986)	0.894 (0.831-0.939)	0.862 (0.792-0.924)
Siemens	130	0.714 (0.554-0.843)	0.989 (0.938-1.000)	0.968 (0.833-0.999)	0.879 (0.798-0.936)	0.942 (0.888-0.984)
GE Vingmed	96	0.787 (0.643-0.893)	0.957 (0.855-0.995)	0.949 (0.827-0.994)	0.818 (0.691-0.909)	0.942 (0.891-0.980)
TOSHIBA	66	0.686 (0.507-0.831)	1.000 (0.888-1.000)	1.000 (0.858-1.000)	0.738 (0.580-0.861)	0.887 (0.774-0.961)
Fujifilm	9	0.750 (0.194-0.994)	1.000 (0.478-1.000)	1.000 (0.292-1.000)	0.833 (0.359-0.996)	0.950 (0.778-1.000)

Abbreviations as in Table 1, Table 2.

a 7 patients did not have BMI available.

## Discussion

In this study, we evaluated the performance of a computer vision–driven AI workflow for the detection and screening of CA across a wide range of patients from an international cohort of geographically distinct health care systems. In this setting, we found EchoNet-LVH had strong performance in identifying patients with CA of all subtypes, and its performance was consistent across sites, ultrasound manufacturers, and patient characteristics.

A few things are worth considering in evaluating our algorithm. Because increased wall thickness is a hallmark of CA,^1^^,^^23^ our algorithm automated the approach to precisely measuring wall thickness as well as integrated this precise measurement with more “black box” features of motion and texture assessed in the apical-4-chamber view. Our approach was designed to minimize or exclude confounders,^13^ as our models were trained on controls matched on wall-thickness and limit the potential of AI models to shortcut on wall thickness alone.

Although EchoNet-LVH performed well across all sites, there was a site-level heterogeneity that might be the result of variations in imaging protocols across countries or patient populations. Further study is needed in larger populations to evaluate these impacts. Our approach sought to maximize PPV in the downstream testing of CA. In a rare disease, PPV is significantly impacted by the model specificity (as the number of false positives is likely to outweigh the number of potential true positives). In the algorithm development, we optimized for specificity as to minimize the number of false positives rejected in downstream testing.

In this study evaluating the performance of an AI algorithm on echocardiogram images, an end-to-end computer vision approach was able to detect ATTR and AL CA from an unselected population of patients 65 years of age or greater undergoing echocardiography. Performance was similar across patient characteristics and geographic sites, suggesting generalizability of a computer vision approach despite differences in practice patterns. If validated in future studies, incorporation of this easily obtainable software evaluation can assist with earlier diagnosis of CA and result in reduced morbidity and mortality.

### Study Limitations

A few limitations are worth considering as further study is still warranted. Prospective trials of CA screening approaches that evaluate clinical outcomes have not yet completed.^24^^,^^25^ There are limited data on the true population prevalence of CA,^3^, ^4^, ^5^^,^^26^ which significantly impacts the PPV of any algorithm in true clinical deployment, and our prior work have shown that choice of controls in retrospective cohorts impact the descriptive model performance.^13^^,^^27^ Other approaches have been proposed to screen for CA;^28^, ^29^, ^30^ however, EchoNet-LVH represent one of the few fully automated pipelines that can augment clinicians without additional data processing or feedback. Furthermore, there is significant observer variability in most echocardiographic measurements,^31^ which limit the precision of approaches based on routine echo measurements alone.^8^ Parallel work has assessed the performance of EchoNet-LVH in comparison to score-based decision aids and electronic health record–based algorithms and showed computer vision–based approaches having superior performance.^30^

Perspectives **COMPETENCY IN MEDICAL KNOWLEDGE:** Recently United States Food and Drug Administration cleared AI algorithm can assist clinicians in identifying patients >65 years of age with cardiac amyloidosis.

**TRANSLATIONAL OUTLOOK:** Additional research is needed to understand impact on clinical workflow in prospective evaluation.

## Funding support and author disclosures

This work is funded by 10.13039/100000002NIH 10.13039/100000050NHLBI grants R00HL157421, R01HL173526, and R01HL173487 to D.O., and a grant from 10.13039/100006396Alexion AstraZeneca Rare Disease. Grant Duffy is currently an employee of Meta. D.O. reports consulting fees and/or equity in Ultromics, InVision, EchoIQ, and Pfizer. Evangelos K. Oikonomou is supported through NIH NHLBI (F32HL170592); he is a co-founder of Evidence2Health LLC, an ad hoc consultant for Caristo Diagnostics Ltd and Ensight-AI Inc; and has received royalty fees from technology licensed through the University of Oxford, outside this work. Rohan Khera reports support from NIH NHLBI (R01HL167858 and K23HL153775), NIH NIA (R01AG089981), and the 10.13039/100000862Doris Duke Charitable Foundation (2022060); he is an Associate Editor of JAMA and receives research support, through Yale, from the Blavatnik Foundation, Bristol-Myers Squibb, Novo Nordisk, and BridgeBio; he is a coinventor of Pending Patent Applications WO2023230345A1, US20220336048A1, 63/346,610, 63/484,426, 63/508,315, 63/580,137, 63/606,203, 63/619,241, and 63/562,335; and a co-founder of Ensight-AI, Inc and Evidence2Health, LLC. Faraz S. Ahmad has received research support from Pfizer and Atman Health and consulting fees from Alnylam Pharmaceuticals. All other authors have reported that they have no relationships relevant to the contents of this paper to disclose.

## References

[1] Writing Committee, Kittleson M.M., Ruberg F.L. (2023). 2023 ACC expert consensus decision pathway on comprehensive multidisciplinary care for the patient with cardiac amyloidosis: a report of the American college of cardiology solution set oversight committee. J Am Coll Cardiol.

[2] Kittleson M.M., Maurer M.S., Ambardekar A.V. (2020). Cardiac amyloidosis: evolving diagnosis and management: a scientific statement from the American heart association. Circulation.

[3] Kim H.M., Sohn D.-W., Paeng J.C. (2019). Prevalence of positive 99 mTc-DPD scintigraphy as an indicator of the prevalence of wild-type transthyretin amyloidosis in the elderly. Int Heart J.

[4] Quock T.P., Yan T., Chang E., Guthrie S., Broder M.S. (2018). Epidemiology of AL amyloidosis: a real-world study using US claims data. Blood Adv.

[5] Treglia G., Martinello C., Dondi F. (2023). Prevalence of incidental findings suspicious for transthyretin cardiac amyloidosis among patients undergoing bone scintigraphy: a systematic Review and a meta-analysis. J Clin Med.

[6] Lee G.Y., Kim K., Choi J.O. (2014). Cardiac amyloidosis without increased left ventricular wall thickness. Mayo Clin Proc.

[7] Cipriani A., De Michieli L., Porcari A. (2022). Low QRS voltages in cardiac amyloidosis: clinical correlates and prognostic value. JACC CardioOncol.

[8] Cotella J., Randazzo M., Maurer M.S. (2024). Limitations of apical sparing pattern in cardiac amyloidosis: a multicenter echocardiographic study. Eur Heart J Cardiovasc Imaging.

[9] Ichikawa Y., Oota E., Odajima S. (2023). Impact of tafamidis on echocardiographic cardiac function of patients with transthyretin cardiac amyloidosis. Circ J.

[10] Maurer M.S., Schwartz J.H., Gundapaneni B. (2018). Tafamidis treatment for patients with transthyretin amyloid cardiomyopathy. N Engl J Med.

[11] Fontana M., Berk J.L., Gillmore J.D. (2024). Vutrisiran in patients with transthyretin amyloidosis with cardiomyopathy. N Engl J Med.

[12] Goto S., Mahara K., Beussink-Nelson L. (2021). Artificial intelligence-enabled fully automated detection of cardiac amyloidosis using electrocardiograms and echocardiograms. Nat Commun.

[13] Duffy G., Cheng P.P., Yuan N. (2021). High-Throughput precision phenotyping of left ventricular hypertrophy with cardiovascular deep learning. arXiv.

[14] Sangha V., Oikonomou E.K., Khera R. (2024). Artificial intelligence applied to electrocardiographic images for scalable screening of transthyretin amyloid cardiomyopathy. medRxiv.

[15] Omori H., Kawase Y., Mizukami T. (2024). Diagnostic performance of artificial intelligence-based angiography-derived non-hyperemic pressure ratio using pressure wire as reference. Circ J.

[16] Omori H., Kawase Y., Mizukami T. (2023). Diagnostic accuracy of artificial intelligence-based angiography-derived fractional flow reserve using pressure wire-based fractional flow reserve as a reference. Circ J.

[17] Yamamoto T., Kawase Y., Mizukami T. (2024). Enhanced plaque stabilization effects of alirocumab - insights from artificial intelligence-aided optical coherence tomography analysis of the alirocumab for thin-cap fibroatheroma in patients with coronary artery disease estimated by optical coherence tomography (ALTAIR) study. Circ J.

[18] Holste G., Oikonomou E.K., Mortazavi B.J. (2023). Severe aortic stenosis detection by deep learning applied to echocardiography. Eur Heart J.

[19] Pirruccello J.P., Bick A., Wang M. (2020). Analysis of cardiac magnetic resonance imaging in 36,000 individuals yields genetic insights into dilated cardiomyopathy. Nat Commun.

[20] Vrudhula A., Duffy G., Vukadinovic M. (2024). High throughput deep learning detection of mitral regurgitation. bioRxiv.

[21] Vukadinovic M., Tang X., Yuan N. (2024). EchoPrime: a multi-video view-informed vision-language model for comprehensive echocardiography interpretation. arXiv.

[22] Akerman A.P., Porumb M., Scott C.G. (2023). Automated echocardiographic detection of heart failure with preserved ejection fraction using artificial intelligence. JACC Adv.

[23] Pagourelias E.D., Mirea O., Duchenne J. (2017). Echo parameters for differential diagnosis in cardiac amyloidosis. Circ Cardiovasc Imaging.

[24] Gouverneur C. (2024). BridgeBio partners with leading cardiovascular data science lab (CarDS lab) to improve transthyretin amyloid cardiomyopathy (ATTR-CM) diagnosis in diverse patient populations with multimodal artificial intelligence. BridgeBio. https://bridgebio.com/news/bridgebio-partners-with-leading-cardiovascular-data-science-lab-cards-lab-to-improve-transthyretin-amyloid-cardiomyopathy-attr-cm-diagnosis-in-diverse-patient-populations-with-multimodal-artificia/.

[25] National Library of Medicine. Artificial Intelligence Guided Echocardiographic Screening of Rare Diseases (EchoNet-Screening). https://clinicaltrials.gov/study/NCT05139797.

[26] Gilstrap L.G., Dominici F., Wang Y. (2019). Epidemiology of cardiac amyloidosis–associated heart failure hospitalizations among fee-for-service medicare beneficiaries in the United States. Circ Heart Fail.

[27] Vrudhula A., Stern L., Cheng P.C. (2024). Impact of case and control selection on training artificial intelligence screening of cardiac amyloidosis. JACC Adv.

[28] Huda A., Castaño A., Niyogi A. (2021). A machine learning model for identifying patients at risk for wild-type transthyretin amyloid cardiomyopathy. Nat Commun.

[29] Davies D.R., Redfield M.M., Scott C.G. (2022). A simple score to identify increased risk of transthyretin amyloid cardiomyopathy in heart failure with preserved ejection fraction. JAMA Cardiol.

[30] Hourmozdi J., Easton N., Benigeri S. (2024). Evaluating the performance and potential bias of predictive models for the detection of transthyretin cardiac amyloidosis. medRxiv.

[31] Pillai B., Salerno M., Schnittger I., Cheng S., Ouyang D. (2024). Precision of echocardiographic measurements. J Am Soc Echocardiogr.

